# Graphitic carbon nitride nanosheet electrode-based high-performance ionic actuator

**DOI:** 10.1038/ncomms8258

**Published:** 2015-06-01

**Authors:** Guan Wu, Ying Hu, Yang Liu, Jingjing Zhao, Xueli Chen, Vincent Whoehling, Cédric Plesse, Giao T. M. Nguyen, Frédéric Vidal, Wei Chen

**Affiliations:** 1 i-Lab, Suzhou Institute of Nano-tech and Nano-bionics, Chinese Academy of Sciences, Suzhou 215123, P. R. China; 2 Laboratoire de Physicochimie des Polymères et des Interfaces, Institut des Matériaux, Université de Cergy-Pontoise, 5 mail Gay-Lussac, 95031 Cergy-Pontoise Cedex, France

## Abstract

Ionic actuators have attracted attention due to their remarkably large strain under low-voltage stimulation. Because actuation performance is mainly dominated by the electrochemical and electromechanical processes of the electrode layer, the electrode material and structure are crucial. Here, we report a graphitic carbon nitride nanosheet electrode-based ionic actuator that displays high electrochemical activity and electromechanical conversion abilities, including large specific capacitance (259.4 F g^−1^) with ionic liquid as the electrolyte, fast actuation response (0.5±0.03% in 300 ms), large electromechanical strain (0.93±0.03%) and high actuation stability (100,000 cycles) under 3 V. The key to the high performance lies in the hierarchical pore structure with dominant size <2 nm, optimal pyridinic nitrogen active sites (6.78%) and effective conductivity (382 S m^−1^) of the electrode. Our study represents an important step towards artificial muscle technology in which heteroatom modulation in electrodes plays an important role in promoting electrochemical actuation performance.

## Abstract (web summary)

Electrochemical actuators store electric energy and generate mechanical motion. Here, the authors present an ionic actuator based on a hierarchically porous graphitic carbon nitride electrode, which exhibits high charge storage, fast actuation response, large electromechanical strain and high stability.

## Graphical abstract

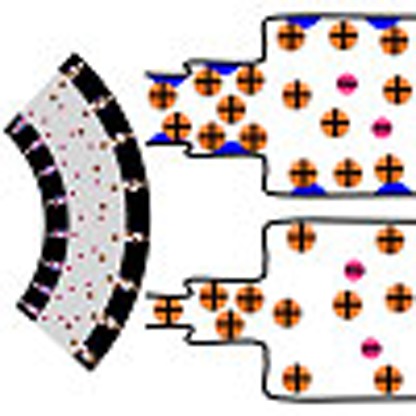

Electrochemical actuators that can store electric energy and convert it into mechanical motion are being extensively studied due to the fast-growing demand for potential applications, such as artificial muscles, biomimetic flying insects, portable consumer devices and micro- and nanoelectromechanical systems^1^,^2^,^3^,^4^,^5^,^6^. Among the various electrochemical actuators, ionic polymer metal composite (IPMC) actuators, which are composed of a layer of polymer electrolyte sandwiched between electrodes, have emerged as promising candidates due to their large actuation deformation and air working stability under low voltage^7^,^8^,^9^. They store electrical energy in a double interface and convert it into mechanical output by reversible ion intercalation and deintercalation of electrodes^10^,^11^. The electrodes, including like low-dimensional carbon materials with porous networks, large specific surface areas (SSAs) and high electrical conductivity, which preserve larger capacitance and allow more ion storage, present high-performance electrochemical–mechanical behaviour^12^,^13^,^14^,^15^,^16^. For example, Asaka's group utilized high aspect ratio millimetre-long single-walled carbon nanotubes (ML-SWCNTs) as actuator electrodes. The superb mechanical and electrical properties of the millimetre-long single-walled carbon nanotubes films gave the IPMC actuators much faster strain and stress rates^17^. In addition, three-dimensional nanocarbon electrodes (RGO-CNTs) with highly stable porous networks exhibited promising cycling stability (one million cycles) in electrochemical actuators^18^. Furthermore, anisotropic arranged vertically aligned carbon nanotube (VA-CNTs) electrodes, which provided continuous ionic conductive path channels for ion fast transport and created desired elastic anisotropy to enhance the strain induced along the actuation direction, presented extremely large actuation strain^19^.

To improve the chemical activity, active materials such as metal oxide (RuO_2_^20^ and MnO_2_^21^) and conducting polymer (PANI^22^ and PPy^23^), were added to nanocarbon electrodes through physical sonication, and the actuation performance was improved by increasing the capacitance. However, the bad miscibility of the active materials in electrodes seriously damaged the electrode's conductivity and interface charge transfer. Therefore, a challenge remains to create nanocarbon electrodes that not only utilize their intrinsic properties (porous network, large SSA and high conductivity), but also endow electrochemical activity. Recent experiments show nitrogen doping can improve the electrochemical activity of nanocarbon materials and thus enhance device performance in various applications, including electrocatalysts, supercapacitors, biosensors and Li-ion batteries^24^,^25^. Vijayaraghavan *et al.*^26^ reported that Pt particles supported by N-doped CNTs showed enhancement of catalytic activity and durability along with N-dopant contents. Reddy *et al.*^27^ demonstrated that the reversible discharge capacity of N-doped graphene was nearly doubled compared with pristine graphene due to the dense surface defects. Among various electrochemical functions of nitrogen doping, a key feature is that nitrogen doping can modify the local electronic structure to increase the surface charge density^28^ and the interaction with ions^29^, which may directly favour enhancement of the electrochemical strain level of ionic actuators. However, there are no reports of N-doped carbon electrode-based electrochemical actuators. In addition, there has been little substantial progress in elaborating the relation between electrochemical processes to the mechanical bending motion of ionic actuators. This implies that the improvement of the electrochemical activity of carbon electrodes by nitrogen doping and understanding how the nitrogen doping influences the mechanical bending motion of the actuators are necessary to identify the role of electrochemical activation in promoting the development of electrochemical actuation performance.

In the present study, motivated by the physicochemical effects caused by nitrogen doping, we develop an ionic actuator based on a hierarchically porous graphitic carbon nitride (g-CN) nanosheet electrode. To enhance the structural stability of the electrodes, a small portion of carbon nanotubes (CNTs) was added to the g-CN network. To provide sufficient ions for rapid migrating, an interpenetrating polymer network based on poly(ethylene oxide) and nitrile butadiene rubber (PEO-NBR) is introduced, which has proven to be a promising electrolyte layer with higher ion uptake, ionic conductivity and ion transportation^14^,^30^,^31^. Our designed actuator displays high charge storage and electromechanical conversion abilities, including large specific capacitance with EMIBF_4_ as the electrolyte (259.4 F g^−1^, seven times higher than pure two-dimensional (2D) graphene electrode), fast actuation response (0.5±0.03% in 300 ms) caused by rapid charge injection, and large equilibrium electromechanical strain under 3 V (up to 0.93±0.03%, three times higher than graphene electrodes). The improvement of the electromechanical motion attributed to the N-active site that increases the charge density as well as enhances the binding interaction with cations and the hierarchical pore structure with dominant size <2 nm has a leading effect on the electrode volume expansion. We believe that our results will play a guiding role in the design of electrochemically active electrode materials to achieve a higher performance electrochemical actuator.

## Results

### Preparation and characterization of g-CN

**Figure 1:**
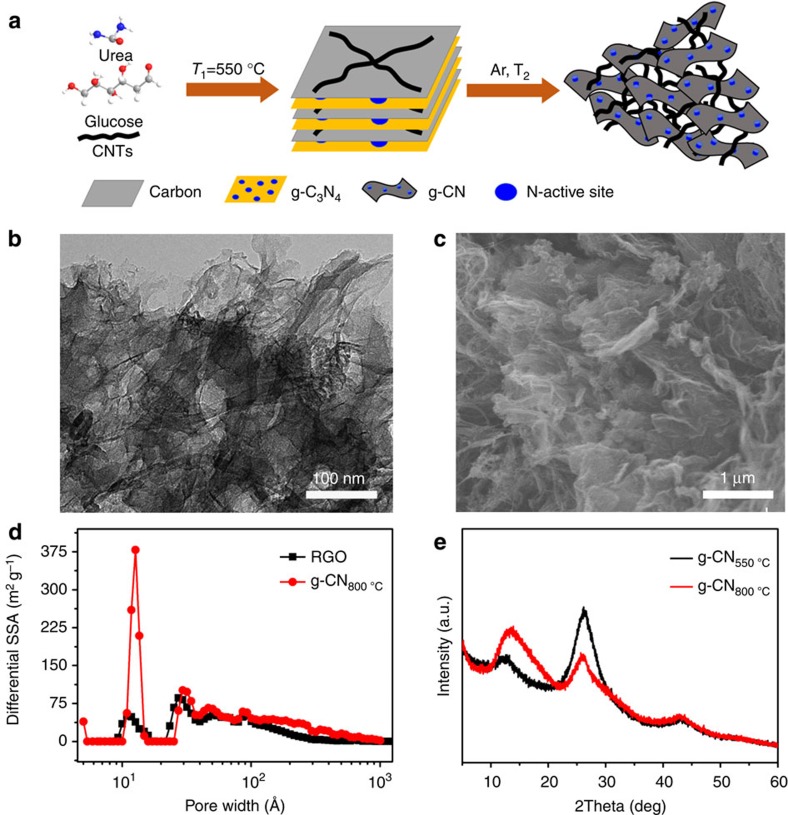
Fabrication of the porous graphic carbon nitride electrode. (**a**) Illustration of the preparation of the g-CN materials. (**b**) TEM images of g-CN_800 °C_, scale bar 100 nm. (**c**) SEM images of g-CN_800 °C_, scale bar 1μm. (**d**) The pore size distribution of g-CN_800 °C_ and RGO and their contribution to SSA. (**e**) XRD patterns of g-CN_550 °C_ and g-CN_800 °C_.

A two-step method is introduced to synthesise g-CN by the calcination of a mixture of glucose, urea and CNTs (Fig. 1a and Supplementary Fig. 1). In first step, thermal condensation of urea created graphite carbon nitride (g-C_3_N_4_), and glucose formed aromatic carbon intermediates on its surface by means of donor–acceptor interactions and finally confined the condensation in a cooperative process to the interlayer gaps of g-C_3_N_4_ to form the derived carbon nanosheets^32^. Then, in the second step, under an Ar atmosphere, the g-C_3_N_4_ served as a sacrificial template and underwent complete thermolysis at 800 °C to provide a nitrogen source for the glucose-derived carbon nanosheets^33^ and graphitic carbon nitride sheets were obtained. The morphology of the as-prepared g-CN_800 °C_ was revealed by transmission electron microscopy (TEM) and scanning electron microscopy (SEM). The TEM image (Fig. 1b) shows well-connected g-CN_800 °C_ nanosheets coupled with CNTs. Fig. 1c further demonstrates a loose lamellar and ribbon-like structure, which exhibits much more porosity than the widely investigated reduced graphene oxide (RGO) in Supplementary Fig. 2. In the overall formation process, the CNTs not only enhanced the mechanical strength and electrical conductivity between layers of the carbon nitride nanosheets through *π*–*π* interaction, but also prevented g-CN from restacking to obtain a high specific surface area^34^.

The porous characteristics are clearly revealed by the N_2_ adsorption isotherm measurement. The whole pore size distributions and their contribution to the SSA from micro, meso to macropore are shown in Fig. 1d. Compared with RGO, g-CN_800 °C_ exhibits a sharp peak at ∼1.27 nm, which is attributed to the interaction between g-CN_800 °C_ and CNTs. Due to these dominated micro and mesopores, especially those with a diameter <2 nm, the SSA of g-CN_800 °C_ (284.3 m^2^ g^−1^) is considerably larger than that of RGO (108.91 m^2^ g^−1^). The exact pore size contributed by SSA is illustrated in Supplementary Table 1. X-ray diffraction is used to characterize the 2D layered structure of g-CN_800 °C_. As shown in Fig. 1e, the presence of g-C_3_N_4_ can be verified with peaks at 26.14° and 13.10°, which originated from the (002) interlayer diffraction of the CN graphitic-like structure and in-planar repeating units with a period of 0.675 nm, respectively^35^. After the sample was treated at 800 °C, the peak at 26.22° gradually became much weaker, whereas the peak at ∼13° became much stronger, indicating a recovery of the in-planar structure. X-ray photoelectron spectroscopy shows a typical N 1s peak and primary graphitic C 1s peak^33^,^36^,^37^. The N 1s signal can be deconvoluted into four subpeaks at approximately 396.3, 399.7, 400.9 and 402.8 eV, which correspond to pyridinic N, pyrrolic N, graphitic N and oxygenated N, respectively (see Supplementary Fig. 3a). In addition, five peaks can be fitted in the C 1s spectrum: the peak located at ∼284.5 eV corresponds to graphitic bonding of sp^2^ C=C; the peak at 285.7 eV can be attributed to sp3 C and C=N; the peak at 287.2 eV is caused by the presence of C=N/C=O; the peak at 288.3 is identified as O–C=O; and the peak at 290.5 eV corresponds to N–C=O (see Supplementary Fig. 3b).

### Construction of g-CN electrode-based actuators

**Figure 2:**
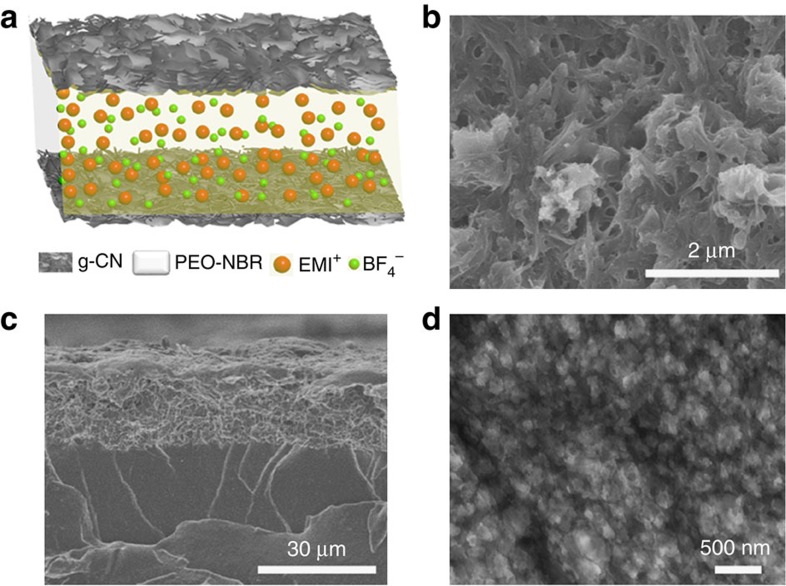
Construction of the graphic carbon nitride electrode-based electrochemical actuator. (**a**) Preparation of the g-CN_800 °C_ actuator. SEM images of the surface, scale bar 2 μm (**b**) and cross section, scale bar 30 μm (**c**) of the g-CN_800 °C_ actuator. (**d**) TEM image of PEO-NBR, scale bar 500 nm.

The new actuator (see Fig. 2a) was fabricated by binding two pieces of as-synthesized free-standing g-CN_800 °C_ films (prefilled ionic liquids in g-CN network) with an electrolyte layer of PEO-NBR supporting EMIBF_4_ via hot press (details are given in the Methods section). As observed in Fig. 2b,c, the porous g-CN_800 °C_ electrode with good electric conductivity (382 S m^−1^) had a good interlayer adhesion with the PEO-NBR layer. The interpenetrating polymer network has successfully been chosen as ion uptaking and transportation supporting in conducting polymer actuators^30^,^38^. In this work, PEO-NBR is used, for the first time, in carbon-based ionic actuators. After comprehensive consideration of the ionic conductivity and mechanical properties, we chose a weight ratio of PEO and NBR of 60/40 as the middle layer. At room temperature (25 °C), our PEO-NBR middle layer exhibited not only high measured ionic liquid uptake capability (70%), but also high measured ionic conductivity (2.5 × 10^−4^ S cm^−1^), which makes PEO-NBR a prime electrolyte layer for faster ionic transportation than other ion-conductive electrolyte membranes^31^. TEM observations were recorded on OsO_4_-loaded PEO-NBR stained samples^39^. Because only the butadiene segments of the NBR could be preferentially marked by OsO_4_, the black domains could be interpreted as NBR-rich phases; thus, PEO-rich phases appeared as clear-grey domains. The TEM image of PEO-NBR shown in Fig. 2d demonstrated a well-distributed PEO phase in an NBR matrix. Taking full consideration of the electrode structure and ion properties of the electrolyte layer, our actuator benefits significantly from this designed architecture. First, the porous g-CN network with a large ion-accessible surface and good electric conductivity will greatly facilitate more charge accumulation in the electrodes. Second, the high ionic conductivity and ionic liquid uptake capability in PEO-NBR promote ion migration ability and increase the time–response process in the electrochemical actuator. Therefore, our electrochemical actuator exhibits good charge storage and electromechanical output behaviour.

### Charge storage in g-CN actuators

**Figure 3:**
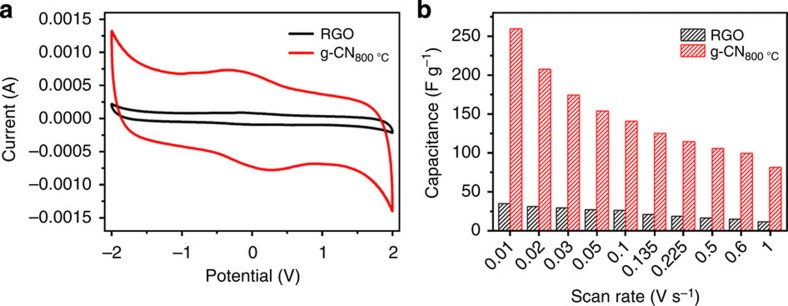
Charge storage behaviour of the electrochemical actuators. (**a**) Cyclic voltammetry (CV) curves for the g-CN_800 °C_ and RGO electrode-based actuators at 10 mV s^−1^ sweep rate. (**b**) The calculated capacitances of devices at different scan rates.

To evaluate the actuators' charge storage and mechanical conversion behaviour, a 20 × 2.5 mm width strip was attached to an electrochemical work station, and the charge storage behaviour of a cyclic voltammeter was employed to evaluate it. As illustrated in Fig. 3a, the porous g-CN_800 °C_ actuator shows a better charging and discharging behaviour than the widely used RGO actuator. At a scan rate of 10 mV s^−1^, the specific capacitance of g-CN_800 °C_ was 259.4 F g^−1^, which was more than seven times larger than RGO (34.9 F g^−1^). This remarkable result could be attributed to the porous network with a large ion-accessible surface and the nitrogen-doping-modified electronic structure of the carbon sheets. The capacitive value was comparable to those of graphene-based flexible supercapacitors^29^,^34^,^40^,^41^. There was a redox pair in the CV curve of the electrochemical actuator because of the reaction between pyridinic nitrogen and water^42^. At the higher scan rate of 100 mV s^−1^, the g-CN_800 °C_ actuator still preserved a nearly rectangular charge/discharge property, indicating a good capacitive behaviour and efficient electrolyte ion transport throughout the electrodes (see Supplementary Fig. 4), and the capacitance was calculated as 140.8 F g^−1^. The detailed capacitance variation was plotted in Fig. 3b. Over all current densities, the g-CN_800 °C_ actuator showed better capacitance than the RGO-based actuator.

### Actuation performance of the g-CN actuator

**Figure 4:**
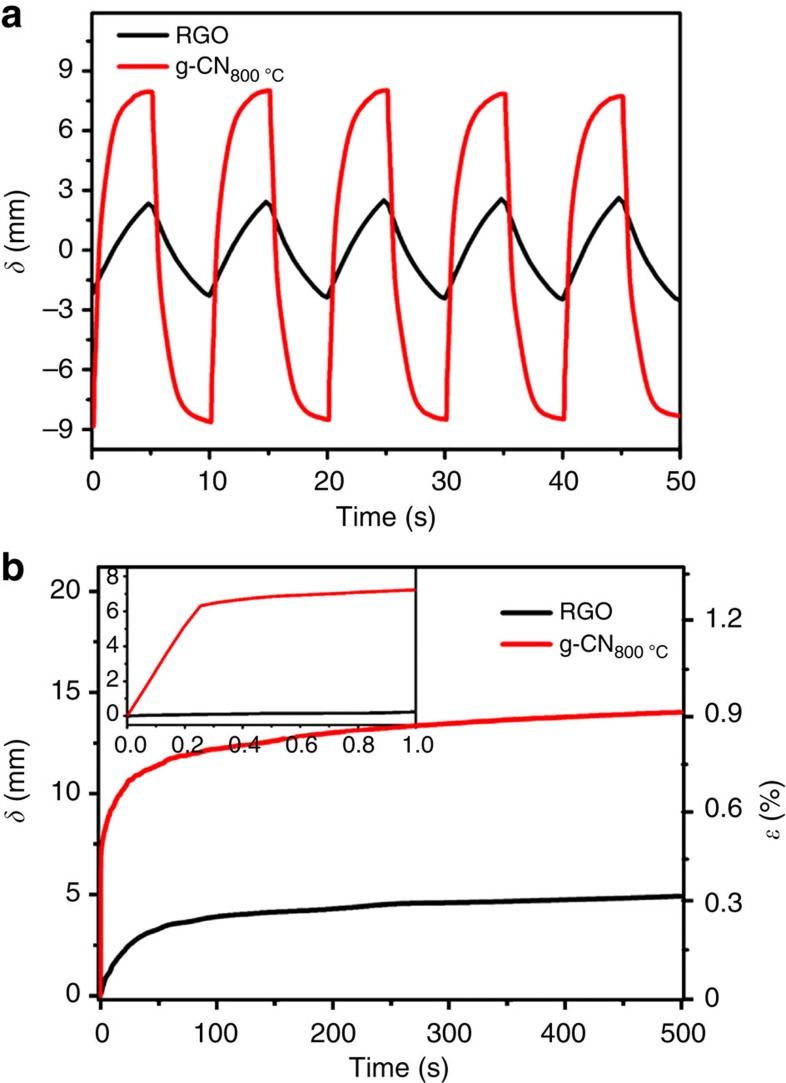
Bending performance of low-voltage-driven electrochemical actuators. (**a**) Bending response of actuators based on g-CN_800 °C_ and RGO electrodes at an applied square wave voltage of ±3 V with a frequency of 0.1 Hz. (**b**) Time-dependent displacement of g-CN_800 °C_ and RGO actuators under 3 V.

The electromechanical bending motion of the g-CN_800 °C_ actuator under different frequencies of electrical stimulation of ±3 V square wave voltage was investigated. The bending motion (*δ*) was monitored by a laser displacement metre. As shown in Fig. 4a, Supplementary Fig. 5a and Supplementary Movie 1, the g-CN_800 °C_ actuator displayed large average peak to peak bending displacement (average deviation 16.45±1.05 mm) at a frequency of 0.1 Hz, which was several times higher than the pure RGO actuator (4.5±0.23 mm) and other reported graphene hybrid-based actuators^18^,^43^,^44^,^45^,^46^. The g-CN_800 °C_ actuator could also maintain good stability without any notable deterioration over 100,000 times continuous operation in air (Supplementary Fig. 6). Moreover, the response rate of the g-CN_800 °C_ actuator was much faster than that of the RGO actuator. The time-dependent displacement curves in Fig. 4b and Supplementary Fig. 5b show that the g-CN_800 °C_ actuator generated large displacement and strain (6.09±0.57 mm, 0.5±0.03%) in only 300 ms (see inset of Fig. 4b and Supplementary Fig. 5b) and reached the largest displacement and strain (14.34±0.46 mm, 0.93±0.03%) in ∼500 s. The fast and slow actuation processes were attributed to the quick insertion of prefilled ions into the dominant small pores (Fig. 1d) and the diffusion of ions from the electrode–electrolyte interface or PEO-NBR polymer afterwards. However, due to the restacked structure, the RGO electrode-based actuator only displayed slow deformation processes (4.73±0.21 mm, 0.32±0.01% in ∼500 s). The porous g-CN_800 °C_ performed larger bending deformation than the restacked RGO actuator (see Supplementary Fig. 7) and even larger than CNTs and RGO/CNTs composite electrode-based actuators in a wider frequency range (∼0.1–10 Hz) (see Supplementary Fig. 8). In addition, the frequency-dependent actuation displacement of the actuator was in good accordance with its electrochemical charging injection (see Supplementary Fig. 9). The mechanical output forces were also tested, and the results are provided in Supplementary Fig. 10; we concluded that the blocking forces increase with decreasing input frequency. Our actuator maintained a relatively low blocking force (0.93 mN under 4 V, 0.01 Hz), and we will further improve its level in our future work. On the basis of the basic bending motion (Supplementary Fig. 11), we obtained actuators of linear motion (Supplementary Fig. 12 and Supplementary Movie 2) and S-type movement (Supplementary Fig. 13 and Supplementary Movie 3).

### N-doping dependent charge storage and mechanical output

**Figure 5:**
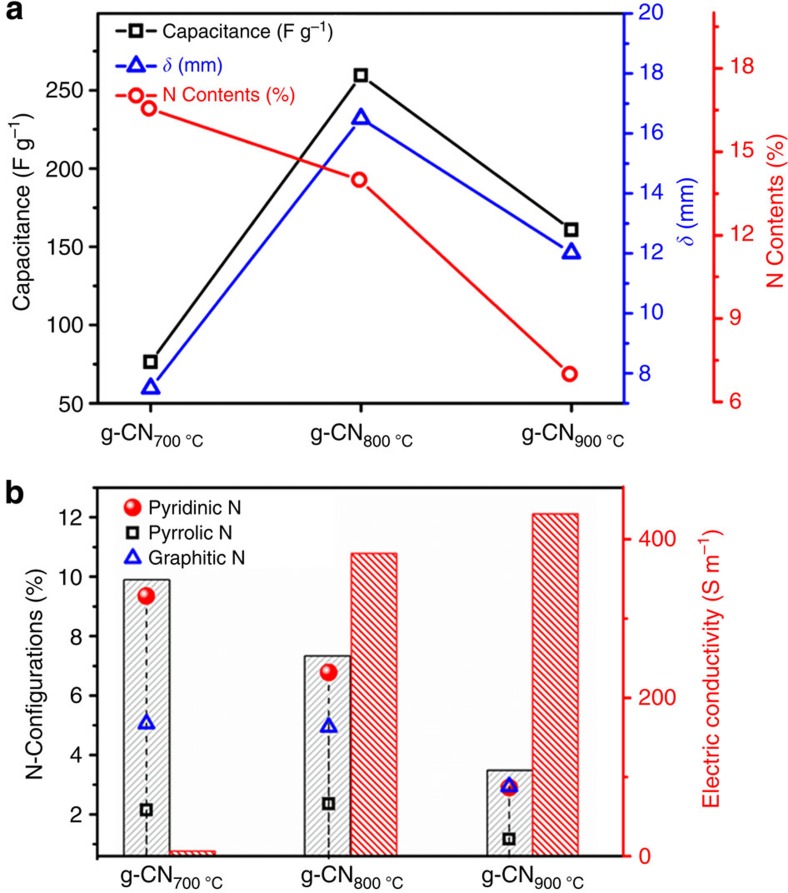
Nitrogen active site analysis. (**a**) The calculated capacitances (scan rate 10 mV s^−1^), peak to peak bending displacement (0.1 Hz) and N contents of the g-CN electrodes. (**b**) The contribution of different N active sites and the electric conductivity of the actuators.

To investigate and understand how nitrogen doping affected the electrochemical–mechanical performance of the actuators, g-CN_700 °C_, g-CN_800 °C_ and g-CN_900 °C_ with different nitrogen contents and configurations were investigated for comparison. As described in Fig. 5a, the g-CN_800 °C_ actuator presented the best capacitance (259.4 F g^−1^), compared with the g-CN_700 °C_ (76.25 F g^−1^) and g-CN_900 °C_ (160.88 F g^−1^) actuators, which agreed with their electrochemical strain performance. The diversity in the capacitance and strain level is related to their different nitrogen contents and configurations. X-ray photoelectron spectroscopy showed that the overall nitrogen contents consisted of g-CN_700 °C_ (16.56%), g-CN_800 °C_ (13.99%) and g-CN_900 °C_ (7.01%). This result indicated that the highest N contents of g-CN_700 °C_ exhibited the lowest electrochemical–mechanical performance, which did not agree with the capacitance and mechanical motion in Fig. 5a. To further investigate the result, we analysed the N-configurations (pyridinic N, pyrrolic N and graphitic N) of the g-CN samples (Fig. 5b). Comparing g-CN_800 °C_ with g-CN_700 °C_, the contents of the pyrrolic N and graphitic N were nearly the same, whereas the slightly decreased pyridinic N (6.78%) in g-CN_800 °C_ greatly improved the electrode's conductivity (382 S m^−1^) in contrast to g-CN_700 °C_ (pyridinic N 9.34%, poor conductivity 6.4 S m^−1^). Thus, pyrrolic N balanced the electrochemical activity and electric conductivity of the electrode to improve the capacitance by increasing the surface charge density of the electrode and enhancing the interaction with ions^28^,^29^ When the sample was treated at 900 °C, g-CN_900 °C_ preserved the highest conductivity (432 S m^−1^). The seriously degraded N-active sites substantially decreased the electrochemical activity, resulting in a drop of the capacitance and mechanical motion. Therefore, considering the optimal balance between the electrochemical activities (N-active sites) and conductivity, the g-CN_800 °C_ exhibited the best capacitance and actuation performance.

## Discussion

**Figure 6:**
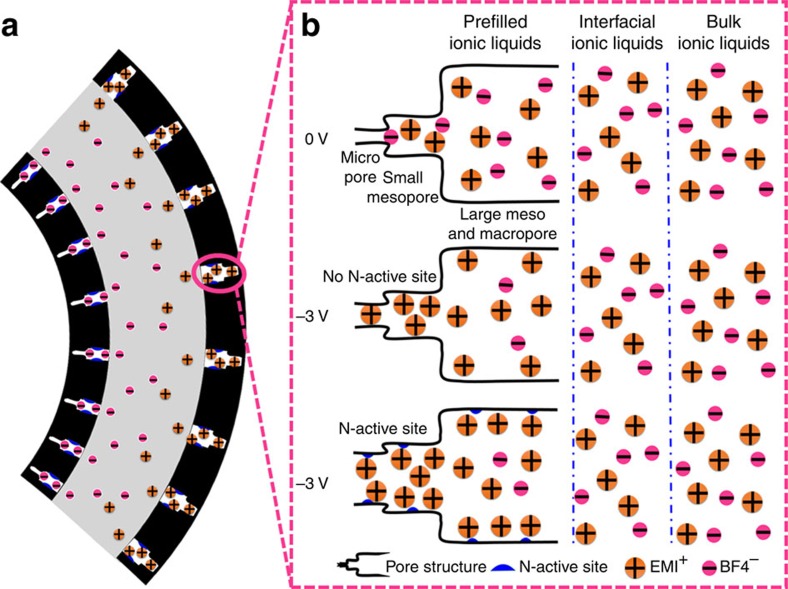
Schematic illustration of the actuation mechanism. (**a**) Bending motion of the g-CN actuator. (**b**) Schematic illustration of the pore structure and cation-induced mechanical output of the g-CN actuator.

In light of the results obtained so far, we speculated the actuation mechanism for the g-CN-based actuator. For carbon electrode-based actuators, when a voltage was applied between the electrode layers, the ions were intercalated into porous electrodes and the volume of electrodes expanded because of the pressure generated inside the electrodes^47^. Because the cations were bigger than the anions, the actuation showed a bending motion^48^. A voltage-induced pressure was generated in terms of the balance between the electrostatic and volume exclusion interactions, which were greatly varied in pore distribution. When the pore size became smaller, the electrostatic interaction became stronger because the distance between the ion and the electrodes became shorter, and the strong electrostatic interaction-caused distance was only several nanometres, whereas the volume exclusion interaction became more significant because the space available for ions became smaller and the number of ions in the pore decreased^47^. Kiyohara *et al.*^49^ comprehensively analysed the voltage-induced pressure in pore distribution that caused deformation of carbon electrode-based actuators. For pore sizes larger than 4 nm, the pressure inside of the porous electrode was virtually the same as in the bulk electrolyte. However, with pore widths <4 nm, there were marked changes of pressure because the effect of the electrostatic interaction was relatively larger than the volume exclusion interaction, indicating that the pores below 4 nm played vital roles in the electrode volume change. Therefore, considering the porous structural characteristics of our materials (see Fig. 1d), we utilized the hierarchically porous model (see Fig. 6) of a macropore sleeve with meso- and micropores to analyse the mechanism of how the charge storage was converted into mechanical motion. As shown in Fig. 6a, when an electric field was applied on the actuator, the ion was intercalated into the porous structure of the electrode, resulting in a bending motion of the actuator. In our actuators, three types of ionic liquids (ILs), prefilled ILs, interfacial ILs and bulk ILs, played different roles in the contribution of mechanical motion (see Fig. 6b). After an electric field was applied on the actuator, the prefilled ion could quickly intercalate into the micro- and small mesopores, and the pore volume expanded, which resulted in a rapidly increasing displacement (300 ms) and diffusion of ions from the electrode–electrolyte interface or PEO-NBR polymer afterwards. When doping nitrogen in carbon materials, the manipulated local electronic structure (N-active site) increased the charge density and enhanced its binding interaction with cations^50^,^51^, which accommodated more cations in pores, and the electrode volume expansion was further increased. Furthermore, all pores contribute to capacitance, but only those pores below 4 nm played a role in electrode volume expansion. g-CN_800 °C_ has considerably more micropores (<2 nm) than RGO, and these pores were closer to the ion sizes, making them more suitable for large volume expansion of the actuator^52^. Therefore, taking full consideration of the hierarchically pore structure and N-active site, the g-CN_800 °C_ actuator displayed a higher actuation performance than the restacked RGO actuator.

In summary, we develop a graphitic carbon nitride electrode-based ionic actuator. The outstanding electrochemical activity and electrical properties of the g-CN electrodes facilitate the highly improved performance of the actuator, including fast actuation response (0.5±0.03% in 300 ms), high actuation stability (100,000 cycles) and large equilibrium electromechanical strain (0.93±0.03%). The high electromechanical motion results from the N-active site increasing the charge density and enhancing the binding interaction with cations, which accommodate more cation intercalation and deintercalation of the electrodes. Additionally, in the hierarchically porous network, micro- and small mesopores make a leading effect to electrode volume expansion (especially those pores <2 nm that are close to the cation size). To the best of our knowledge, this type of heteroatom doped electrochemical actuator has not been reported. Considering these remarkable achievements together with the flexibility, lightweight and large mechanical motion, we believe that the graphitic carbon nitride-based electrochemical actuator will have great potential to meet various biomimetic technology demands.

## Methods

### Synthesis of g-CN

g-CN was synthesized by thermal treatment of 0.5 g of glucose (from Sigma Aldrich), 10 g of urea (AR, Sinopharm Chemical Reagent Co., Ltd) and 0.06 g of SWCNTs (Nanjing XFNANO Materials Tech Co., Ltd). The detailed synthesis process is shown in Supplementary Fig. 1. A two-step method is introduced to synthesize the g-CN electrode. In the first step, all precursors, including urea, glucose and CNTs, were dried at 80 °C for 24 h. Then, the precursors were ground uniformly and put in a crucible (100 ml in volume) with bayonet cap. The crucible was placed in a muffle furnace and heated to 550 °C for 3 h under ambient air. In the second step, the pretreated samples were transferred to a tube furnace with the following programmed heating process: heating at 100 °C for 1 h under Ar protective flow of 200 sccm to remove O_2_ and water vapour and then continued heating at higher temperatures (700, 800 and 900 °C) to obtain different levels of g-CN products.

### Preparation of RGO

GO was prepared by a modified Hummers method, which has been described in our recent work^53^,^54^. GO dispersion was reduced by N_2_H_4_ in a 90 °C oil bath to form an RGO suspension. Next, the suspension was filtrated and washed with deionized water, obtaining RGO samples.

### Preparation of the PEO-NBR/EMIBF_4_ electrolyte layer

PEO/NBR (weight ratio 60/40) films were obtained by an *in situ* polymerization according to a modified previously described procedure^38^. First, the given amounts of NBR were poured into a flask with corresponding amounts of 1,2-trichloroethane. The obtained mixture was stirred for 24 h at room temperature. Then, the given amounts of poly(ethylene glycol) methacrylate methyl ether (PEGM) and poly(ethylene glycol) dimethacrylate (PEGDM) corresponding to the PEO/NBR ratio were added to the mixture and stirred for 15 min at room temperature. In all preparations reported in this study, PEGM and PEGDM were introduced at a constant weight ratio corresponding to 75 wt% PEGM and 25 wt% PEGDM in the PEO network. Dicyclohexylperoxidicarbonate (DCPD) was used for the methacrylate radical initiation, whereas DCP was the vulcanization agent for NBR. The DCPD initiator (3 wt% with respect to the sum of methacrylate oligomers weight) and dicumyl-peroxide (DCP) (2 wt% of NBR weight) were then added to the mixture and stirred for 15 min at room temperature. The mixture was poured into a mould made from two glass plates clamped together and sealed with a Teflon gasket. This mould was then kept at 50 °C for 3 h, post-cured for 1 h at 80 °C and cooled for 30 min at room temperature. The mould was placed into the oven and cured for different vulcanization times/temperatures. After vulcanization, all synthesized films were dried for 24 h under vacuum to obtain the PEO-NBR polymer. Last, by immersing the PEO-NBR polymer in EMIBF_4_ solution at 80 °C for 4 days, 70 wt% EMIBF_4_ (1-ethyl-3 methylimidazolium tetrafluoroborate) uptake in PEO-NBR was obtained.

### Construction of actuators

A mixture of g-CN (75 mg), PVdF (100 mg) and EMIBF_4_ (150 mg) was dispersed in 30 ml of N,N-dimethylacetamide solution for 30 min for 200 W horn sonication treatment (2 s on and 6 s off in an ice water bath). A 3 ml suspension was casted on a 7.5 × 2.5 cm^2^ area glass substrate and was allowed to stand at room temperature for 3 days before being dried at 80 °C for 6 h. The g-CN electrode film was prepared. After that, two pieces of the electrode layers were laminated on the PEO-NBR/EMIBF_4_ layer through a multi-step hot press: first heat pressed at 120 °C for 5 min then at 100 °C for 15 min. Last, we obtained a bimorph membrane (approximately 85 μm thick, 2.5 mm wide and 20 mm long flexible strip). The RGO actuator was fabricated by the same process that dispersed RGO, PVdF and EMIBF_4_ in N-methyl pyrrolidone under 200 W horn sonication treatment (2 s on and 5 s off for 30 min) to form a gel-like suspension. The suspension was cast on a glass substrate, forming an electrode film (electrical conductivity 312.5 S m^−1^), and two pieces of the electrode film were laminated on PEO-NBR/EMIBF_4_ film through hot press to obtain the actuators.

### Mechanical motion tests

The 20 × 2.5 mm sized strips were all analysed in a two-electrode configuration with 16 mm free length from the electrode contacts. The mechanical bending motion was tested by a multipoint step to the flexible strips using a CHI760D electrochemical work station. The displacement (*δ*) of the device was measured by a Keyence LK-G800 laser positioning system, where the strain (ɛ) and strain rate generated (ɛ_r_) in the actuators were estimated by the following equations:^13^,^55^,^56^


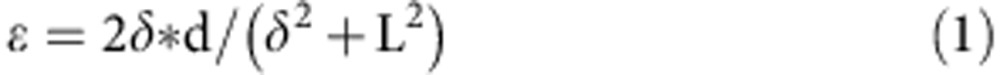


where *d*, *δ* and *L* are the thickness, displacement and free length of the device strip, respectively.

### Other measurements

Sonication was performed using a Fisher Scientific model 500 digital sonic dismembrator equipped with a 12.5 mm diameter disruptor horn. Annealing treatment was conducted using an OTF-1200 tube furnace. FESEM was recorded by a Hitachi S-4800. The electrode conductivity was tested by a multifunction digital four-probe tester (ST-2258C). All electrochemical characterizations were recorded by a CHI760D electrochemical work station. The actuation process and electrochemical characterization were performed in a two-electrode configuration.

## Additional information

**How to cite this article:** Wu, G. *et al.* Graphitic carbon nitride nanosheet electrode-based high-performance ionic actuator. *Nat. Commun.* 6:7258 doi: 10.1038/ncomms8258 (2015).

## Supplementary Material

Supplementary Figures and Table Supplementary Figures 1-13 and Supplementary Table 1

Supplementary Movie 1 Bending motion of actuator under electric stimulus.

Supplementary Movie 2 The linear motion of actuator under electric stimulus.

Supplementary Movie 3 The S-type movement of actuator under electric stimulus.
