# Supplementary material for: Graphitic carbon nitride nanosheet electrode-based high-performance ionic actuator
Source: Nat Commun. 2015 Jun 1;6:7258. doi: 10.1038/ncomms8258 (PMC4458862; doi:10.1038/ncomms8258)
Supplement: Supplementary Figures and Table — Supplementary Figures 1-13 and Supplementary Table 1 [file ncomms8258-s1.pdf]

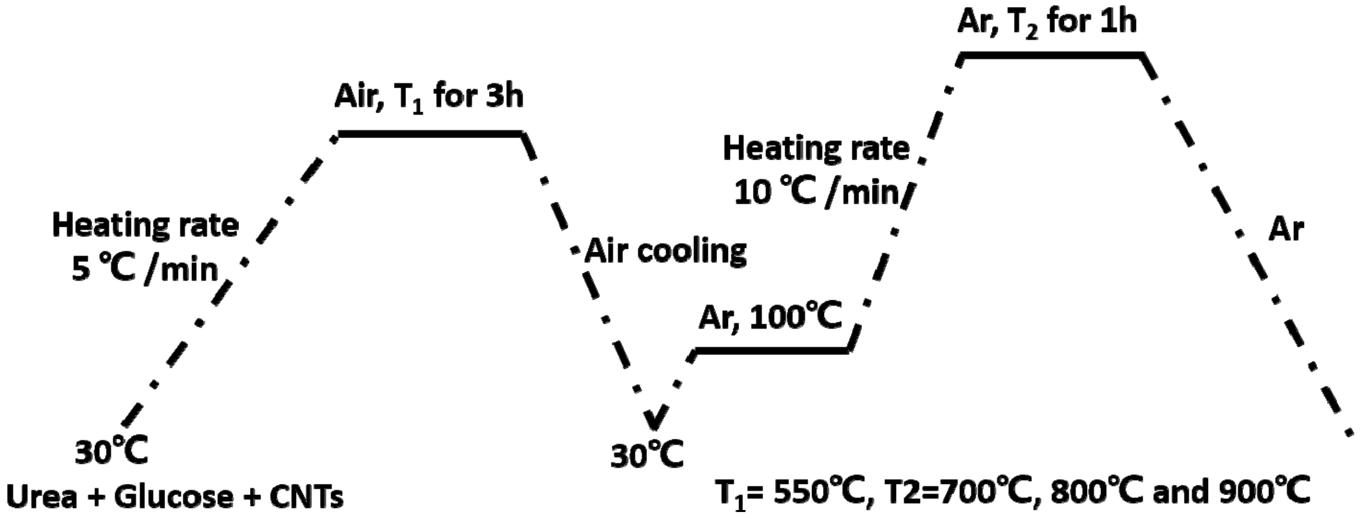

Supplementary Fig. 1 | Process of preparation g-CN. Glucose, urea and CNTs go through a two-step thermal treatment according to the process.

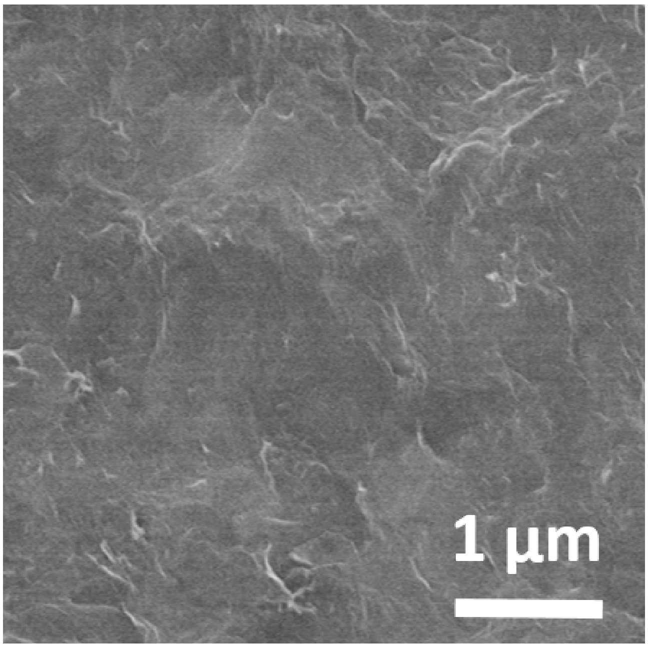

Supplementary Fig. 2 | Surface morphology of RGO. SEM image of RGO, scale bar 1 μm.

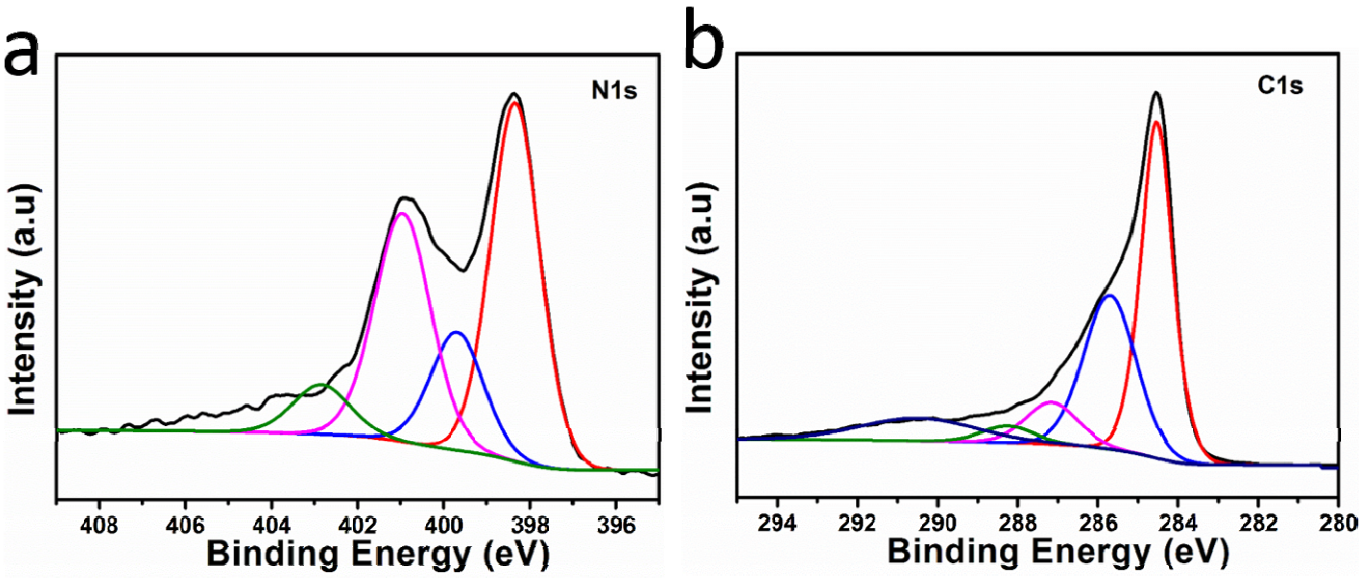

Supplementary Fig.3 | XPS analysis of g-CN. High resolution N1s (a) and C1s (b) XPS spectra of g-$CN_{800℃}$.

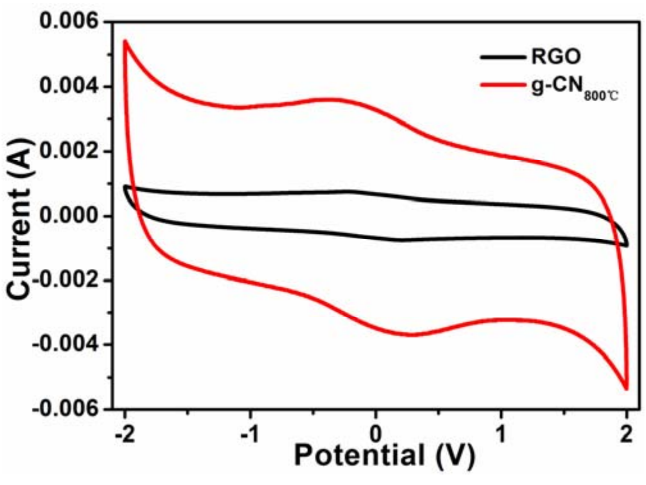

Supplementary Fig. 4 | Charge storage behaviors of electrochemical actuators. Cyclic voltammetry (CV) curves for actuators at 100 mV s^{-1} sweep rate.

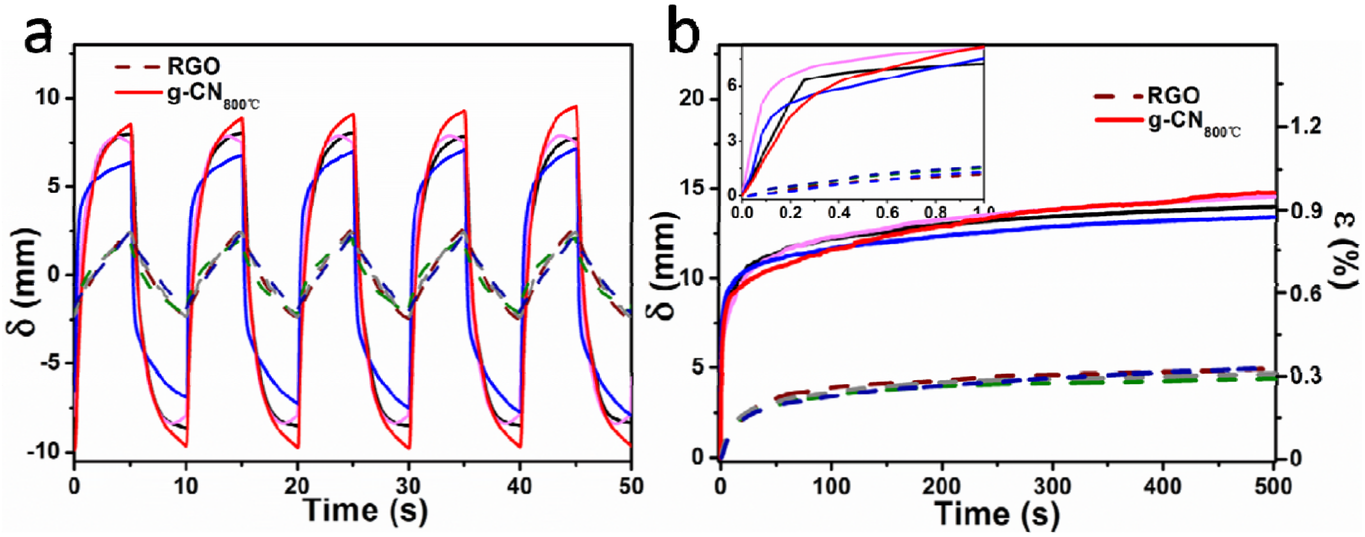

Supplementary Fig. 5 | Bending performance of four pairs of actuator samples. (**a**) Bending response of actuators at ± 3 V, 0.1 Hz. (**b**) Time-dependent displacement of actuators under 3 V. The dash lines stand for RGO actuators and solid lines represent g-$CN_{800℃}$ actuators.

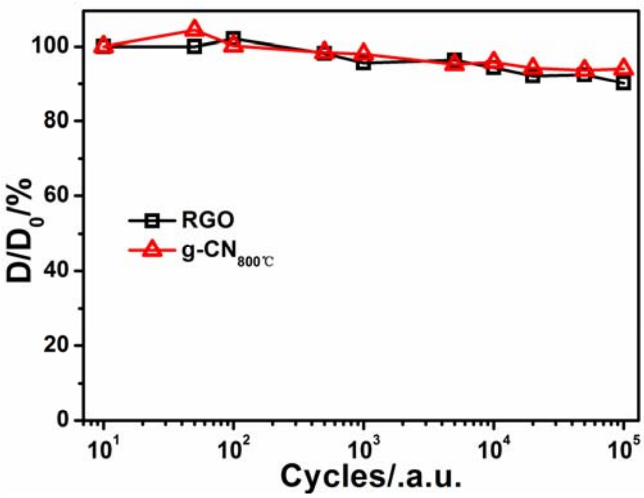

Supplementary Fig. 6 | Actuation stability of electrochemical actuators. Cycling test for RGO and g-$CN_{800℃}$ actuator under ± 3V and 1 Hz applied square wave voltage stimulation, D_0 represents the initial displacement value for tenth cycles.

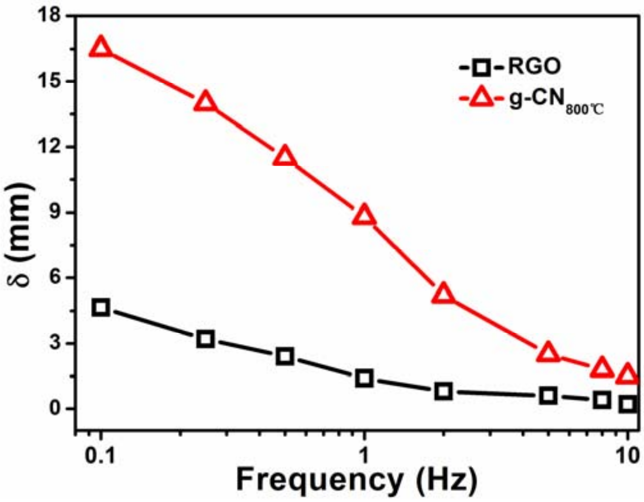

Supplementary Fig. 7 | Wide frequency response of actuators. Actuation performance of wide frequency response (0.1~10 Hz) of RGO and g-$CN_{800℃}$ actuators.

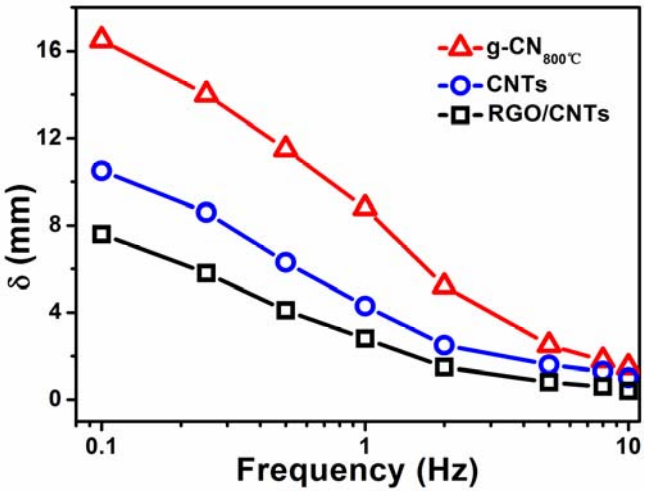

Supplementary Fig. 8 | The comparision of actuation performance. The actuation performance comparison of g-$CN_{800℃}$,CNTs and RGO/CNTs electrode based actuators.

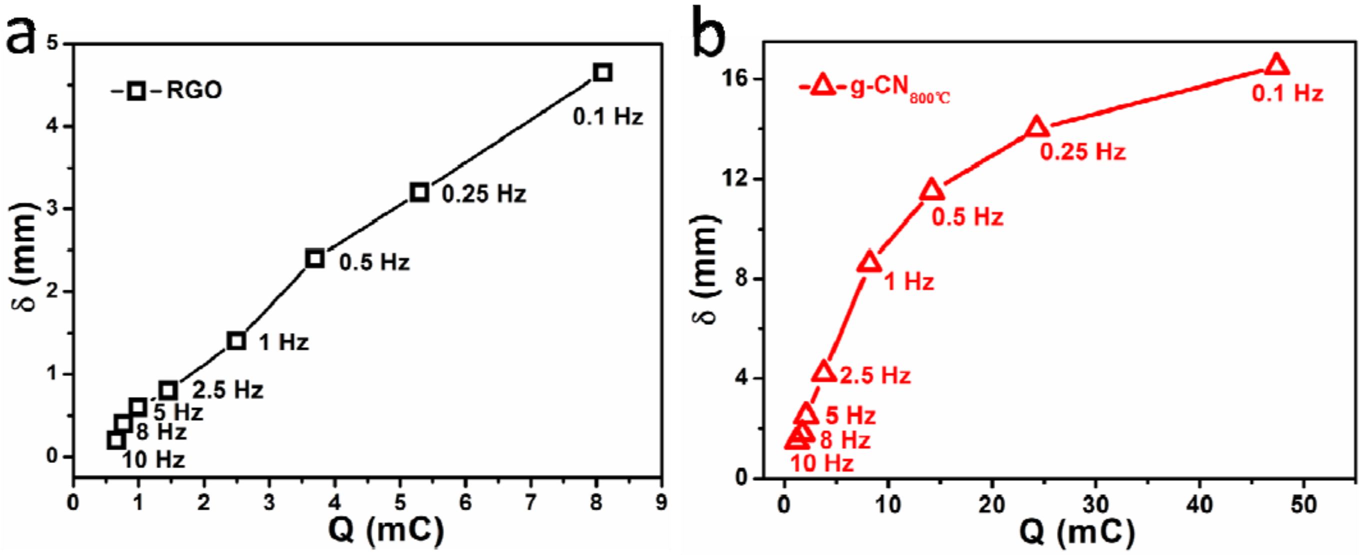

Supplementary Fig. 9 | The relationships between bending displacements and charge injections. Actuation bending displacements along with charge injections under wide frequency response (0.1~10 Hz) of actuators. (**a**) RGO and (**b**) g-$CN_{800℃}$.

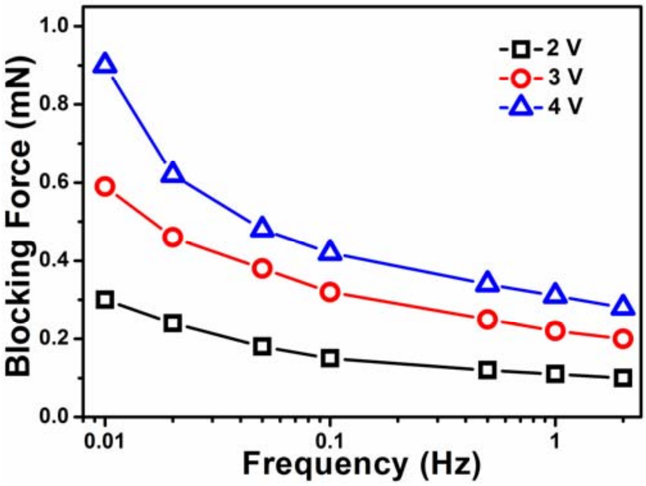

Supplementary Fig. 10 | The mechanical force output of actuator. Blocking force of g-$CN_{800℃}$ actuators under different applied voltages and frequencies stimulation.

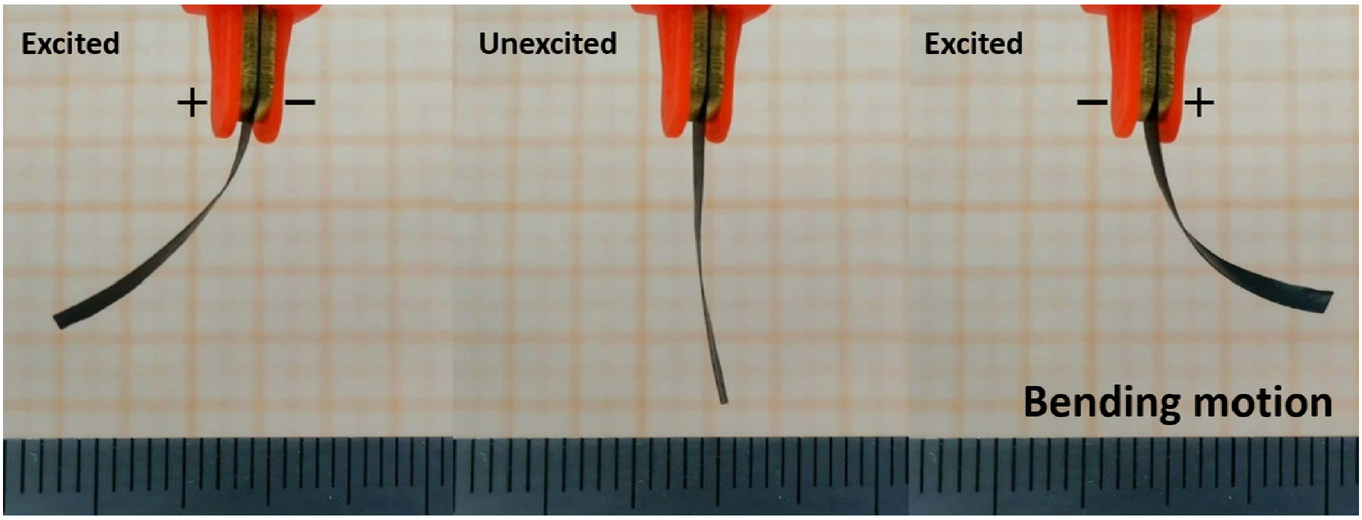

Supplementary Fig. 11 | Bending movement of actuator. Bending motion of g-CN electrode based actuator under the frequency of 0.1Hz.

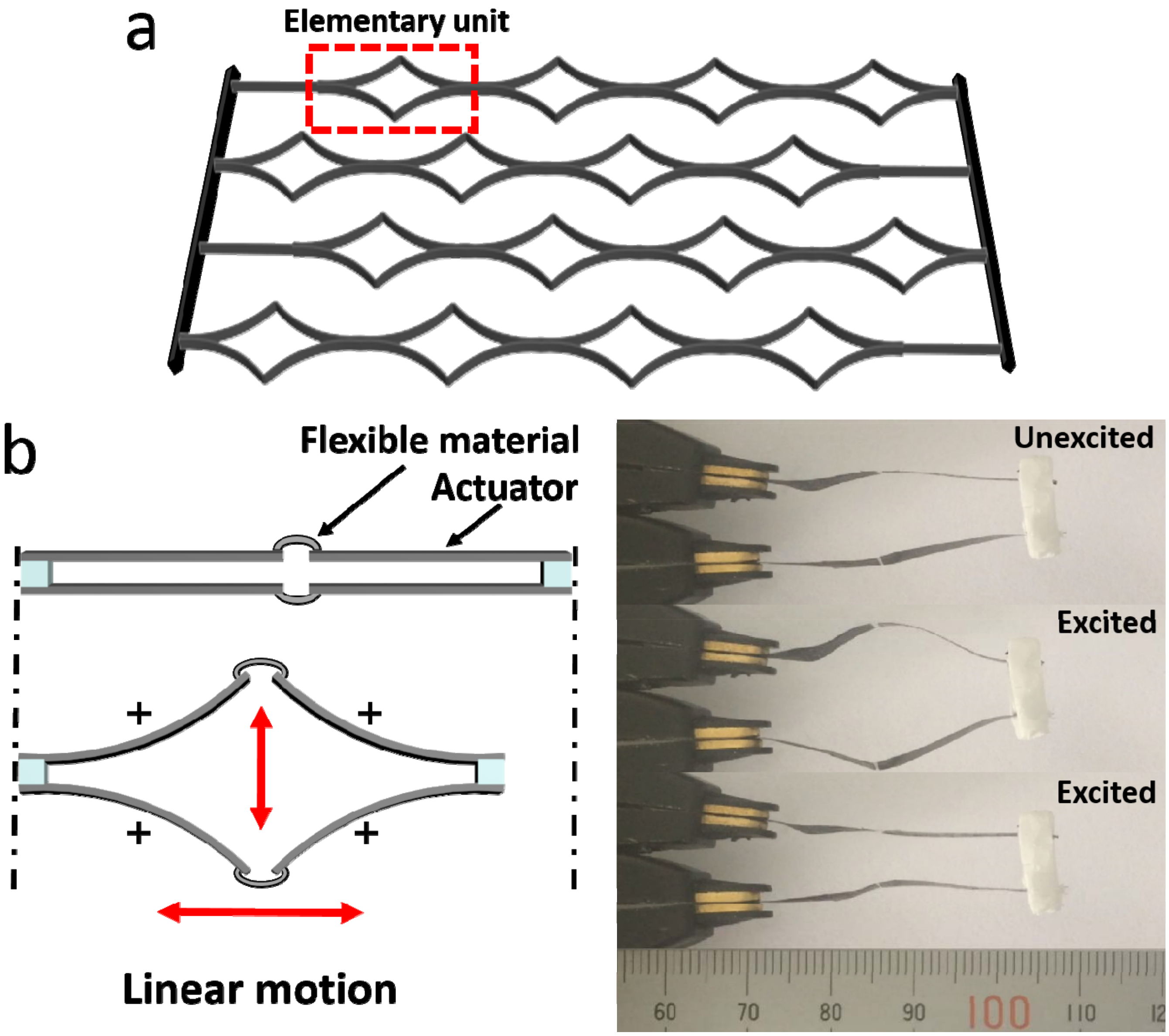

Supplementary Fig. 12 | Schematic illustration of a linear motion actuator. (**a**) Structure of a linear actuator; (**b**) An elementary unit of the proposed linear actuator and its movement under electric stimulus.

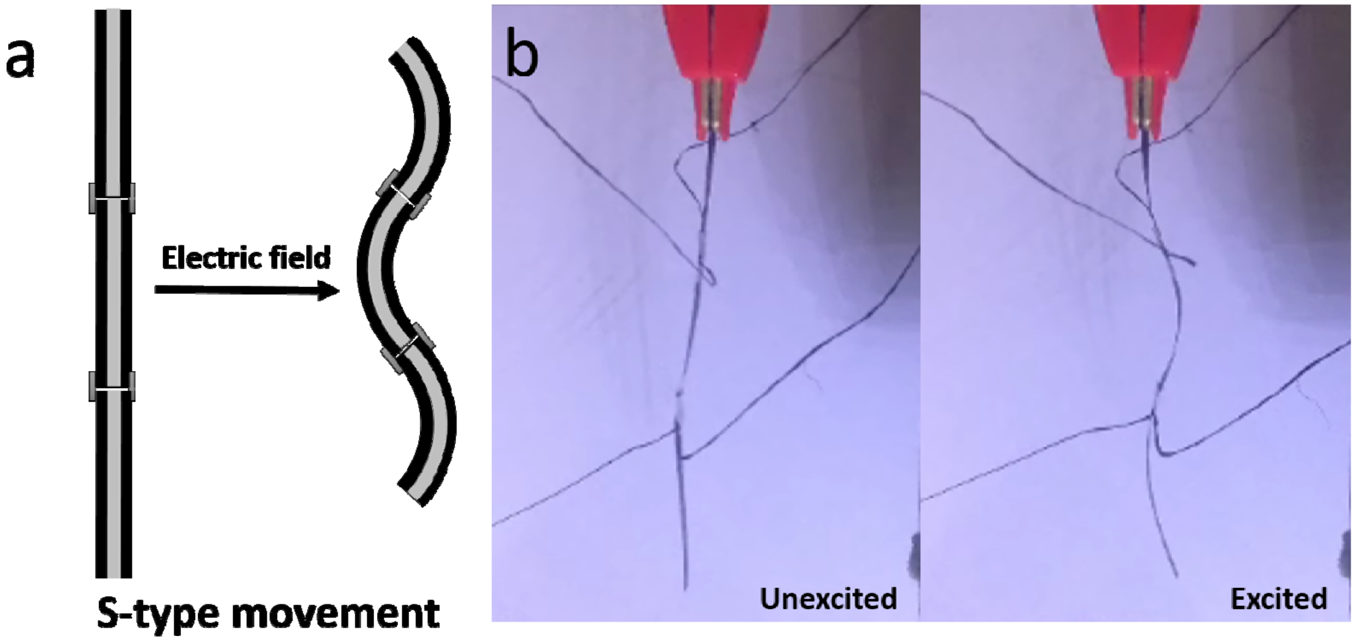

Supplementary Fig. 13 | Schematic illustration of an actuator with S-type movement. (**a**) Structure of the S-type actuator; (**b**) Photographs of the S-type movement actuator under electric stimulus.

Supplementary Table 1 | A summary of pore size distribution and its contribution to SSA

Materials	SSA (m^2g^{-1})	Micropore (%)	Mesopore (%)		Macropore (%)
		<2 nm	2-4 nm	4-50 nm	>50 nm
RGO	108.91	15.24	30.26	53.94	0.56
g-$CN_{800℃}$	284.3	34.49	16.07	47.21	2.23
